# Characteristics of Patients With Hormone Receptor-Positive/Human Epidermal Growth Factor Receptor 2-Negative Early Breast Cancer Eligible for Adjuvant Cyclin-Dependent Kinase 4/6 (CDK4/6) Inhibitors: Real-Life Evidence in a Colombian Cancer Center

**DOI:** 10.7759/cureus.101836

**Published:** 2026-01-19

**Authors:** Alfredo Sebastian Golemba, Pablo Mandó, Adriana Lucia Jimenez Bolaño, Shirly Patricia Rodríguez Torres, Rusvelt Vargas Moranth, Astor Alfonso Aguirre Santamaria

**Affiliations:** 1 Department of Oncology, Centro Cancerológico del Caribe Ltda., Barranquilla, COL; 2 Department of Oncology, Centro de Educación Médica e Investigaciones Clínicas (CEMIC), Buenos Aires, ARG; 3 Department of Statistics, Centro Cancerológico del Caribe Ltda., Barranquilla, COL; 4 Department of Public Health, Universidad del Norte, Barranquilla, COL

**Keywords:** abemaciclib, adjuvant therapies, breast cancer, cancer in latin america, cdk4/6 inhibitors, real-life evidence, ribociclib

## Abstract

Background

The introduction of cyclin-dependent kinase 4/6 inhibitors (CDK4/6i) in the adjuvant setting for hormone receptor-positive (HR+)/human epidermal growth factor receptor 2-negative (HER2-) early breast cancer (BC) has expanded treatment options for high-risk patients. However, real-world data on eligibility for these therapies in Latin American populations remain limited.

Methods

This retrospective study analyzed 334 patients with HR+/HER2- early breast cancer (stages I-III) treated at a Colombian cancer center (2022-2024). Eligibility criteria for adjuvant abemaciclib (monarchE) and ribociclib (NATALEE) trials were applied. Clinicopathological characteristics and treatment patterns were assessed.

Results

Among abemaciclib-eligible patients (36.2%, n=121), 54.5% had ≥4 positive nodes, and 45.5% met alternative high-risk criteria (1-3 nodes plus tumor size of ≥5 cm or grade 3 or Ki-67 of ≥20%). Ribociclib-eligible patients (59%, n=197) included 53.8% with stage III disease and 12.7% with node-negative, grade 3 tumors. Descriptive comparison with pivotal trials shows that compared to pivotal trials, our cohort had an older median age (61 versus 51-52 years) and a lower prevalence of grade 3 tumors (17.1% versus 30%-40%).

Conclusion

A substantial proportion of Colombian patients with HR+/HER2- early breast cancer qualify for adjuvant CDK4/6i, particularly those with nodal involvement. These findings highlight both the clinical opportunity and economic challenges of implementing these therapies in resource-limited settings while underscoring the need for improved early detection to reduce advanced presentations. Eligibility based on clinical-pathological criteria identifies a high-risk group, though optimal selection may require biomarker refinement where available.

## Introduction

Breast cancer (BC) is the most prevalent diagnosed tumor and the principal cause of cancer-related death worldwide and in Colombia among women [1]. It is a heterogeneous disease comprising distinct biological entities with different prognoses [2]. Over 90% of cases are diagnosed at early stages, of which nearly 70% are hormone receptor-positive (HR+)/human epidermal growth factor receptor 2-negative (HER2-) tumors [3,4].

The standard treatment for early-stage HR+/HER2- BC has been adjuvant endocrine therapy (ET) ± chemotherapy (CT) ± radiotherapy over the past 20 years [5]. Despite treatment, patients with early-stage HR+/HER2- BC still face risks of early (≤5 years) and late (>5 years) recurrence, regardless of nodal involvement [6-8]. A meta-analysis showed that women diagnosed with early-stage BC since 2000 have a distant recurrence rate approximately five times lower than those diagnosed in the 1990s [6]. Another real-world study of patients with stage II or III HR+/HER2- BC showed a recurrence risk of 26.1% at five years and 45.0% at 10 years. Patients with stage III disease had a higher risk than those with stage II, as did node-positive compared to node-negative patients. Even those with stage II disease (five-year risk: 22.7%) or negative nodes (five-year risk: 22.1%) still faced a considerable recurrence risk. These findings corroborate the persistent unmet need in this broad population of early-stage HR+/HER2- BC, including those with stage II disease or negative nodes [9].

Cyclin-dependent kinase 4/6 inhibitors (CDK4/6i) have transformed the treatment of luminal metastatic breast cancer by significantly improving progression-free survival (PFS) [10-12] and, in some instances, overall survival (OS) [12]. This success has led to their evaluation in the adjuvant setting for patients with early-stage disease and high-risk features. Clinical trials such as PENELOPE-B [13], PALLAS [14], monarchE [15], and NATALEE [16] have shown promising results for CDK4/6i, though with variability in patient selection criteria and observed benefits, raising questions about optimal patient selection, adverse effect management, and long-term treatment impact.

Several key points must be considered when analyzing this therapeutic strategy. The success of adjuvant CDK4/6i depends on identifying patients most likely to benefit. Features such as positive lymph nodes, high histological grade, and elevated Ki-67 have emerged as relevant factors, but biological and clinical heterogeneity underscores the need for more specific biomarkers. Consequently, the primary objective of this study was to determine the clinical characteristics and distribution of patients with HR+/HER2- BC eligible for abemaciclib and ribociclib in the adjuvant setting.

## Materials and methods

A retrospective, observational, cohort study was conducted at the Centro Cancerológico del Caribe Ltda. in Barranquilla, Colombia. Consecutive patients meeting the inclusion criteria between July 2022 and December 2024 were enrolled. The study included patients over 18 years of age with histologically confirmed HR+/HER2- breast cancer at clinical stages I-III. Patients with HER2-positive breast cancer, HR-negative/HER2-negative disease, advanced-stage cancer, or incomplete medical records for key variables (stage, grade, nodal status, and Ki-67) required for eligibility assessment were excluded. For other variables with missing data (e.g., lymphovascular invasion), the denominator was adjusted accordingly, and data were not imputed. Hormone receptor (HR) positivity was defined as estrogen receptor (ER) and/or progesterone receptor (PR) of ≥1% by immunohistochemistry (IHC). Human epidermal growth factor receptor 2 (HER2) positivity was defined as IHC 3+ or fluorescence in situ hybridization (FISH)-positive (HER2/chromosome 17 centromere {CEP17} ratio of ≥2.0 or average HER2 copy number of ≥6.0 signals/cell). HER2-negative status included IHC 0, 1+, or 2+/FISH-negative (the latter defined as HER2-low). Disease stage was determined according to the American Joint Committee on Cancer (AJCC) eighth edition staging system. Menopausal status was defined clinically: premenopausal as women with regular menses, aged <50 years with prior hysterectomy without bilateral oophorectomy, and postmenopausal as women with ≥12 months of amenorrhea, aged ≥50 years with prior hysterectomy without bilateral oophorectomy or with prior bilateral oophorectomy. For patients who received neoadjuvant chemotherapy (based on the clinical {palpation of pathological lymph nodes, findings compatible with lymph node involvement in axillary ultrasound and/or breast magnetic resonance imaging} and/or pathological {confirmatory biopsy of lymph node involvement of the axilla} condition of the axilla), pathological complete response (pCR) was defined as the absence of invasive carcinoma in the breast and axillary lymph nodes (ypT0/Tis ypN0).

Eligibility was assessed independently for each drug based on the published criteria of the respective pivotal trials [15,16]. For abemaciclib (monarchE criteria), patients were considered eligible if they had (1) ≥4 pathologically positive axillary lymph nodes (pN+) or (2) 1-3 pN+ and at least one of the following: primary tumor size of ≥5 cm (pT3), histological grade of 3, or Ki-67 index of ≥20% [15]. Ki-67 was assessed locally by IHC on diagnostic biopsies or surgical specimens (no data are available regarding the antibody clone and/or staining platform); the reported percentage was based on nuclear staining in invasive tumor cells. For ribociclib (NATALEE criteria), patients were considered eligible if they had stage IIA (with nodal involvement or node-negative with histological grade of 3), stage IIB, or stage III disease [16]. For the subset of patients with stage IIA, node-negative, grade 2 disease, eligibility required Ki-67 of ≥20%. The criterion of "high genomic risk" per the NATALEE protocol was not applied in this analysis due to the unavailability of routine genomic testing in our setting. Nodal and tumor staging was based on the pathological report for patients undergoing upfront surgery or clinical/imaging assessment for those receiving neoadjuvant therapy. Two researchers independently reviewed patient records against these criteria. Discrepancies were resolved by consensus with a senior investigator. A patient could be eligible for both agents.

Clinical-pathological data, including diagnosis date, treatment type, surgical details, and pathology reports, were collected from electronic medical records. The study was approved by the institutional ethics committee and followed Good Clinical Practice guidelines, ensuring adherence to ethical principles and maintaining data confidentiality. This research complied with the guidelines for human studies and was conducted ethically in accordance with the World Medical Association Declaration of Helsinki.

Statistical analysis

Categorical variables were expressed as absolute numbers and percentages. Continuous variables were described in terms of means and standard deviations (SD) if normally distributed or medians and interquartile ranges (IQR) otherwise. Normality was assessed using the Shapiro-Wilk test. Categorical variables were compared using the chi-square or Fisher's exact test, as appropriate. Continuous variables were compared using the independent samples t-test (normal distribution) or Mann-Whitney U test (non-normal distribution). Exact two-sided P-values are reported; P<0.05 was considered statistically significant. Comparisons with the populations of the pivotal clinical trials (monarchE and NATALEE) are presented descriptively. Statistical analysis was performed using R software version 4.3.1 (R Foundation for Statistical Computing, Vienna, Austria). No inferential statistical comparisons were performed. The results are entirely descriptive, and no P-values are reported.

## Results

Patient characteristics

**Figure 1:**
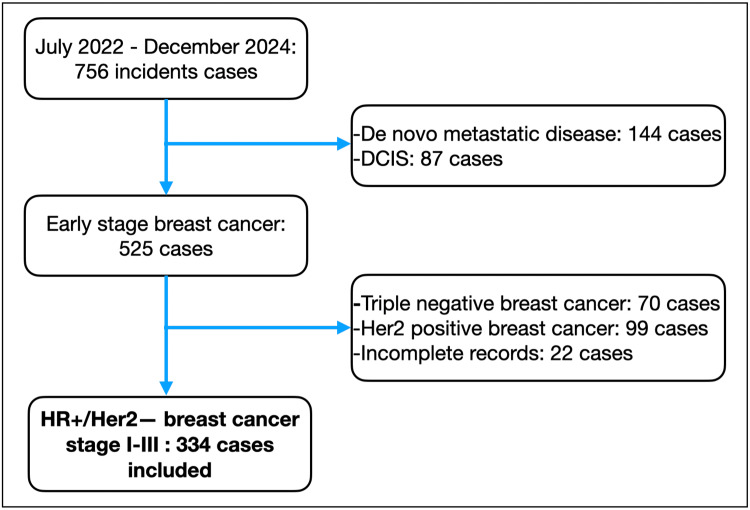
Study flowchart of patients who met the inclusion/exclusion criteria and the final analytical cohort. HR+, hormone receptor-positive; HER2-, human epidermal growth factor receptor 2-negative; DCIS, ductal carcinoma in situ

During the study period, 334 patients met the inclusion criteria (see Figure 1 for study flowchart). The median age was 61 years (IQR: 51-68), with 24.7% (83/334) of the patients diagnosed before age 50. The clinical characteristics of the overall population are detailed in Table 1.

**Table 1:** Characteristics of the population of patients with HR+/HER2- breast cancer. ER-low means estrogen receptor (ER) of <10%; HER2-low means HER2 1+ or 2+ with negative ISH. *n=316; **n=308; ***n=311; ****n=128. SLNB, sentinel lymph node biopsy; ALND, axillary lymph node dissection; pCR, pathological complete response; NOS, not otherwise specified; IQR, interquartile range; HR+, hormone receptor-positive; HER2-, human epidermal growth factor receptor 2-negative; ISH, in situ hybridization

Characteristics	% (N=334)	
Age, years old (median-IQR)	61 (51-68)	
Menopausal status	Premenopausal	22.8 (76)
	Postmenopausal	77.2 (257)
Histology	Ductal/NOS	83.2 (277)
	Lobular	5.4 (18)
	Others	11.4 (38)
Histological grade*	I	30.1 (95)
	II	56.3 (178)
	III	13.6 (43)
Lymphovascular invasion**		17.2 (53)
ER-low	3.3 (11)	
HER2-low	23.4 (78)	
Ki-67≥20%	Yes	45.2 (151)
	No	54.8 (183)
Stage	IA	25.1 (84)
	IIA	26.3 (88)
	IIB	16.8 (56)
	IIIA	16.8 (56)
	IIIB	7.8 (26)
	IIIC	7.2 (24)
Chemotherapy	Adjuvant	7.8 (26)
	Neoadjuvant	43.4 (145)
	None	48.8 (163)
Type of surgery***	Lumpectomy+SLNB	21.2 (66)
	Lumpectomy+ALND	8.4 (26)
	Mastectomy+SLNB	10.6 (33)
	Mastectomy+ALND	59.8 (186)
pCR****	Yes	13.3 (17)

Eligibility for adjuvant abemaciclib

**Figure 2:**
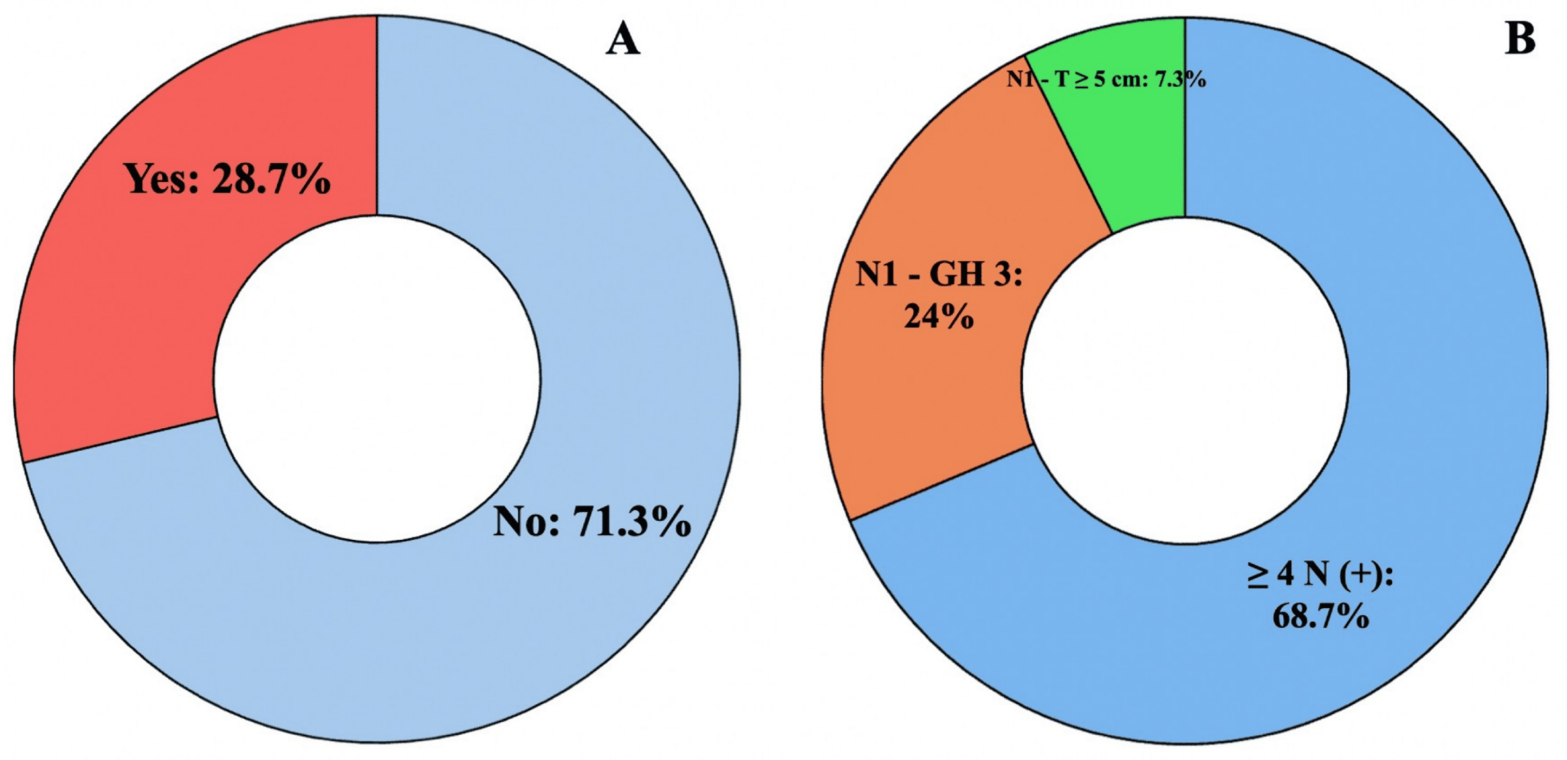
(A) Patients eligible for adjuvant abemaciclib (cohort 1); (B) distribution of patients according to risk factors.

**Figure 3:**
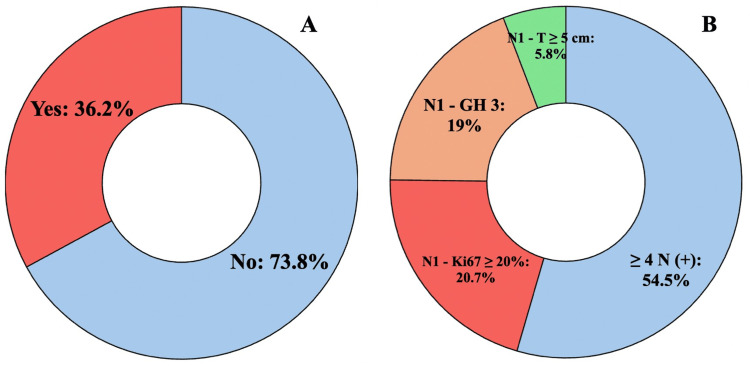
(A) Patients eligible for adjuvant abemaciclib (cohorts 1 and 2); (B) distribution of patients according to risk factors.

As shown in Figure 2A, the evaluation of adjuvant abemaciclib eligibility revealed that 28.7% (96/334) qualified according to cohort 1 criteria from the monarchE study. Figure 2B demonstrates that among these eligible patients, 68.7% (66/96) met the criteria due to N2 or greater nodal involvement. When expanding eligibility to include both study cohorts (Figure 3), the proportion of candidates for adjuvant abemaciclib increased to 36.2% (121/334).

Eligibility for adjuvant ribociclib

**Figure 4:**
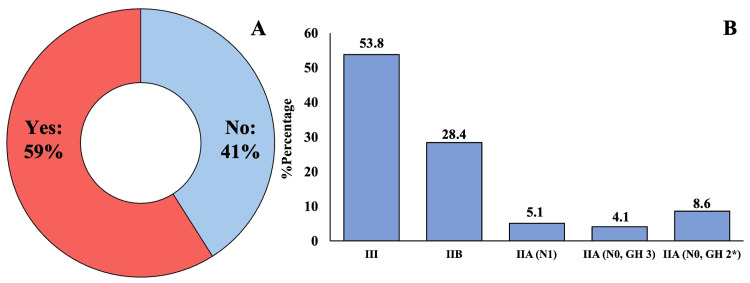
(A) Patients eligible for adjuvant ribociclib; (B) distribution according to the stage of disease. *Ki-67: ≥20%.

Figure 4A presents the analysis of ribociclib eligibility, showing that 59.0% (197/334) would qualify when applying the broader NATALEE trial criteria. As illustrated in Figure 4B, while the majority of eligible patients (53.8%, 106/197) had stage III disease, a notable proportion (12.7%, 25/197) were node-negative.

Comparison with pivotal trial populations

Comparative analysis with pivotal trials (Table 2) demonstrated important demographic differences in our cohort. Patients in our study were older (median age: 58 versus 60 years) and included fewer premenopausal women (25.0% versus 23.0%) compared to the monarchE and NATALEE populations. Tumor characteristics showed a lower percentage of grade 3 tumors among abemaciclib candidates (17.1% versus trial data), while ribociclib-eligible patients had a similar prevalence of grade 3 tumors (17.6% versus trial data).

**Table 2:** Characteristics of patients eligible for adjuvant CDK4/6 inhibitors compared descriptively to the populations of the pivotal trials. Note: Comparisons are descriptive; formal statistical testing between our cohort and trial populations was not performed. ER-low means estrogen receptor (ER) of <10%; HER2-low means HER2 1+ or 2+ with negative ISH. NOS, not otherwise specified; IQR, interquartile range; HER2, human epidermal growth factor receptor 2; ISH, in situ hybridization; CDK4/6, cyclin-dependent kinase 4/6

		monarchE [15], %	Eligible for Abemaciclib (n=121), %-n	NATALEE [16], %	Eligible for Ribociclib (n=197), %-n
Age years old (median-IQR)		51 (23-89)	58 (49-68)	52 (24-90)	60 (50-69)
Premenopausal		43.5	24.8 (30)	43.9	22.8 (45)
Postmenopausal		56.5	75.2 (91)	55.7	77.2 (152)
Histology	Ductal/NOS	-	91.7 (111)	73.3	89.3 (175)
	Lobular	-	5 (6)	17.7	4.1 (8)
	Others	-	3.3 (4)	7.5	6.6 (13)
Histological grade	I	7.4	22.2 (26)	9.0	21.8 (41)
	II	48.9	60.7 (71)	57.0	60.6 (114)
	III	38.8	17.1 (20)	21.0	17.6 (33)
Lymphovascular invasion		-	29 (31)	-	26 (46)
ER-low		-	4.1 (5)	-	4.6 (9)
HER2-low		-	28.1 (34)	-	24.4 (48)
Ki-67≥20%		44.9	26.4 (32)	-	38.1(75)
Stage	IIA	11.5	4.1 (5)	19.6	17.8 (35)
	IIB	13.9	19.8 (24)	20.5	28.4 (56)
	IIIA	36.6	45.5 (55)	59.6	28.4 (56)
	IIIB	3.7	10.7 (13)		13.2 (26)
	IIIC	33.8	19.8 (24)		12.2 (24)
Chemotherapy	Neoadjuvant	37.0	81 (98)	42.7	69.5 (137)
	Adjuvant	58.5	12.4 (15)	47.9	11.7 (23)
	None	4.5	6.6 (8)	11.9	18.8 (37)

These findings, as visualized in Figures 2-4, indicate that 36.2% (121/334) to 59.0% (197/334) of our real-world population would qualify for adjuvant CDK4/6 inhibition under the current criteria. The graphical representation in Figure 3 demonstrates how eligibility expands when considering both cohorts, while Figure 4 highlights the impact of including lower-risk patients in the NATALEE criteria. The results underscore important differences between trial populations and real-world patient demographics, particularly regarding age distribution (24.7% <50 years versus trial populations) and tumor grade (17.1%-17.6% grade 3 tumors), as detailed in Table 2.

## Discussion

This retrospective study evaluated the proportion of patients with early-stage HR+/HER2- breast cancer who met the criteria for adjuvant therapy with CDK4/6 inhibitors at an oncology center in Colombia. The findings reveal that a significant percentage of these patients would be candidates for abemaciclib or ribociclib, with important clinical, economic, and healthcare access implications for the Colombian health system. These results align with international reports, though with regional particularities reflecting disparities in breast cancer diagnosis and management in Latin America.

When comparing our results with pivotal clinical trials, relevant differences emerge. The monarchE and NATALEE studies established eligibility criteria based on high-risk factors such as extensive nodal involvement, tumor size, high histological grade, and elevated Ki-67 [15,16]. However, our cohort showed a distinct distribution of clinical characteristics. The median age was higher than in the reference studies, with a lower proportion of premenopausal patients. Additionally, the percentage of grade 3 tumors was lower than reported in clinical trials, which may reflect differences in pathological assessment or tumor biology in our population.

A notable finding was the high percentage of patients eligible for ribociclib, including a subgroup with stage II disease and negative nodes. This contrasts with the more restrictive monarchE criteria and suggests that in real-world clinical practice, a considerable proportion of intermediate-risk patients could be considered for adjuvant CDK4/6 inhibitor therapy. However, the lack of genomic testing platforms in our setting limits precise risk stratification, which could lead to overtreatment in some cases.

Chemotherapy use in our cohort also differed from that observed in clinical trials. While most patients in monarchE and NATALEE received adjuvant chemotherapy, our study showed lower use of this modality, particularly among abemaciclib candidates. This may be explained by the high percentage of patients presenting with locally advanced disease at the time of diagnosis who received neoadjuvant chemotherapy, as well as by the barriers to access to systemic treatment in our context.

When comparing our results with other real-world studies, similarities and divergences emerge. International retrospective series have reported that between 14% and 45% of patients meet the criteria for adjuvant abemaciclib, depending on population characteristics. Ladoire et al. reported in a retrospective analysis of 3103 patients that 14.2% would be eligible for adjuvant abemaciclib (63.4% due to ≥4 involved axillary nodes) and 34.4% for ribociclib (unlike our population, only 9% had stage III disease) [17]. Tarantino et al. communicated results from a real-world analysis of 7060 American patients, where 14.5% met the criteria for adjuvant abemaciclib and 30.6% for adjuvant ribociclib. Unlike our cohort, only 5% had N2 and N3 axillary involvement [18]. Kanjanapan et al., in a retrospective analysis of 3840 women from an Australian real-world registry, reported that 17.5% met requirements for adjuvant abemaciclib and 41.3% for adjuvant ribociclib [19]. López et al. described a cohort of 107 women, of whom 45% met the criteria for adjuvant abemaciclib; similar to our cohort, 54% had ≥4 involved nodes. A limitation of this analysis was its restriction to premenopausal women only [20]. Jhon Bolaños et al. presented recurrence data from a retrospective cohort of 2142 patients from one of Colombia's major health insurers with stage II and III HR+/HER2- breast cancer. In contrast to our cohort, 68.7% had stage II and 31.3% stage III disease; 12.3% had N2 and N3 disease [21]. Our findings fall at the higher end of this range, possibly due to the high percentage of patients with locally advanced disease at diagnosis in our setting. This situation reflects deficiencies in early detection programs and barriers to oncology service access in Colombia, particularly in the subsidized health system population, which represents most of our patients.

The implications of these findings are multifaceted. Clinically, the high percentage of CDK4/6 inhibitor candidates presents therapeutic decision-making challenges, especially considering these drugs' adverse effects and quality-of-life impact. Eligibility does not equal access, especially in Colombia's stratified health system, which is a mixed model of universal insurance, regulated by the Ministry of Health and Social Protection, which seeks coverage for all through contributory and subsidized regimes (which represent the majority of patients treated at our center). Furthermore, the financial sustainability of incorporating these therapies in Colombia's health system requires rigorous cost-benefit analysis, given CDK4/6 inhibitors' high prices and limited resource availability.

Finally, our results highlight the need for more effective breast cancer early detection strategies in Colombia. The high percentage of patients with locally advanced disease at diagnosis suggests failures in screening programs and timely healthcare access. Community education initiatives, expanded mammography coverage, and reduced administrative barriers could help decrease the proportion of patients requiring intensive adjuvant therapies.

Study limitations include its retrospective, single-center design from a specific Colombian region, which may introduce selection bias and limit generalizability. The lack of centralized review for parameters such as Ki-67 and histological grade may have caused tumor classification variability, affecting the accurate identification of CDK4/6 inhibitor candidates. Furthermore, given the lack of routine access to genomic testing platforms, the application of the "high genomic risk" criterion of the NATALEE trial and more precise molecular risk stratification may influence the underestimation of eligible patients. Another important limitation is the short follow-up period, which precluded the evaluation of clinical outcomes such as disease-free survival associated with these treatments. Finally, as a referral center, the higher proportion of locally advanced patients may not fully reflect breast cancer epidemiology in other Colombian healthcare settings. These limitations underscore the need for multicenter prospective studies incorporating more sophisticated risk stratification tools and longer-term clinical outcome assessments.

## Conclusions

In conclusion, this study provides real-world evidence about the proportion of patients with HR+/HER2- breast cancer who would be CDK4/6 inhibitor candidates in the adjuvant setting in Colombia. One suggestion could be to prioritize CDK4/6 inhibitors (both for abemaciclib and ribociclib) only in those patients who have ≥4 positive nodes. The findings emphasize the importance of optimizing selection criteria, improving access to risk stratification tools, and strengthening early detection programs. Future research should evaluate these therapies' actual impact on survival and quality of life in our setting, as well as their cost-effectiveness in Colombia's health system.

## References

[1] Bray F, Laversanne M, Sung H, Ferlay J, Siegel RL, Soerjomataram I, Jemal A (2024). Global cancer statistics 2022: GLOBOCAN estimates of incidence and mortality worldwide for 36 cancers in 185 countries. CA Cancer J Clin.

[2] Tsang JY, Tse GM (2020). Molecular classification of breast cancer. Adv Anat Pathol.

[3] Howlader N, Altekruse SF, Li CI, Chen VW, Clarke CA, Ries LA, Cronin KA (2014). US incidence of breast cancer subtypes defined by joint hormone receptor and HER2 status. J Natl Cancer Inst.

[4] Cardoso F, Spence D, Mertz S (2018). Global analysis of advanced/metastatic breast cancer: decade report (2005-2015). Breast.

[5] Loibl S, André F, Bachelot T (2024). Early breast cancer: ESMO Clinical Practice Guideline for diagnosis, treatment and follow-up. Ann Oncol.

[6] Early Breast Cancer Trialists' Collaborative Group (2024). Reductions in recurrence in women with early breast cancer entering clinical trials between 1990 and 2009: a pooled analysis of 155 746 women in 151 trials. Lancet.

[7] Pedersen RN, Esen BÖ, Mellemkjær L (2022). The incidence of breast cancer recurrence 10-32 years after primary diagnosis. J Natl Cancer Inst.

[8] Curigliano G, Ciruelos E, Kalinsky K (2024). Short-term risk of recurrence in patients (pts) with HR+/HER2− early breast cancer (EBC) treated with endocrine therapy (ET) in randomized clinical trials (RCTs): a meta-analysis. J Clin Oncol.

[9] O'Shaughnessy J, Tolaney SM, Yardley DA (2025). Real-world risk of recurrence and treatment outcomes with adjuvant endocrine therapy in patients with stage II-III HR+/HER2- early breast cancer. Breast.

[10] Slamon DJ, Diéras V, Rugo HS (2024). Overall survival with palbociclib plus letrozole in advanced breast cancer. J Clin Oncol.

[11] Goetz MP, Toi M, Huober J (2024). Abemaciclib plus a nonsteroidal aromatase inhibitor as initial therapy for HR+, HER2- advanced breast cancer: final overall survival results of MONARCH 3. Ann Oncol.

[12] Hortobagyi GN, Stemmer SM, Burris HA (2022). Overall survival with ribociclib plus letrozole in advanced breast cancer. N Engl J Med.

[13] Loibl S, Martin M, Bonnefoi H (2025). Final survival results from the PENELOPE-B trial investigating palbociclib versus placebo for patients with high-risk HR+/HER2- breast cancer and residual disease after neoadjuvant chemotherapy. Ann Oncol.

[14] Gnant M, Dueck AC, Frantal S (2022). Adjuvant palbociclib for early breast cancer: the PALLAS trial results (ABCSG-42/aft-05/Big-14-03). J Clin Oncol.

[15] Johnston SR, Harbeck N, Hegg R (2020). Abemaciclib combined with endocrine therapy for the adjuvant treatment of HR+, HER2-, node-positive, high-risk, early breast cancer (monarchE). J Clin Oncol.

[16] Slamon D, Lipatov O, Nowecki Z (2024). Ribociclib plus endocrine therapy in early breast cancer. N Engl J Med.

[17] Ladoire S, Kamga AM, Galland L (2025). Prevalence, clinicopathologic features and long-term overall survival of early breast cancer patients eligible for adjuvant abemaciclib and/or ribociclib. NPJ Breast Cancer.

[18] Tarantino P, Rugo HS, Curigliano G (2025). Characteristics of real-world NATALEE- and monarchE-eligible populations with HR+/HER2- early breast cancer: a United States Electronic Health Records database analysis. ESMO Open.

[19] Kanjanapan Y, Anderson W, Smith M, Green J, Chalker E, Craft P (2025). Real-world analysis of breast cancer patients qualifying for adjuvant CDK4/6 inhibitors. Clin Breast Cancer.

[20] Lopez V, Nico A, Jove F, Aguilar A, Cáceres V (2023). Incidence and clinicopathological characteristics of premenopausal patients with early luminal breast cancer candidates for adjuvant therapy with abemaciclib (Article in Spanish). Oncol Clin.

[21] Jhon Bolaños MC, Bello C, Benavides C (2025). Abstract P1-11-23: risk of disease recurrence among patients with stage II and III HR-positive, HER2-negative breast cancer in Colombia: a retrospective cohort study. Clin Cancer Res.

